# The Book World of Medicine and Science

**Published:** 1897-03-13

**Authors:** 


### The Book World of Medicine and Science.

Exposures of Quackery: Being a Series of Articles Upon and Analyses of Various Patent Medicines. By the Editor of "Health News." (London: The Savoy Press. Vols. I. and II., 124 pp. and 141 pp, Price 1s. each.)

The articles contained in these volumes are entitled to a high place in the endeavour which has recently been taking place to move public opinion on the subject of so-called patent medicines. It is to be hoped that the issue of the second volume and a still wider circulation will open the eyes of the public to the many abuses connected with the sale of these quack nostrums. The powers claimed for some of them in the glowing advertisements penned by their proprietors are so little short of miraculous that it is not surprising that they should be believed by the credulous public to contain substances unknown to the medical profession or only to be procured with difficulty. A perusal of these volumes will quickly show how unwarranted are these expectations; the medicinal substances used by quacks being few in number and the active ingredients, as a rule, having been long familiar even to the general public and within the reach of all, for their cheapness as compared with the price of the preparation is a striking characteristic. In addition to the results of analyses by competent chemists of many of these delusive preparations much curious information may be found as to quacks and their ways, and the books should be of value to those interested in the subject, while their untechnical style renders them suitable for popular use.

The Year Book of Treatment for 1897. (London: Cassell and Co. Price 7s. 6d.)

It seems hardly necessary to say much in regard to this well-known annual, which has steadily worked itself to the front during the thirteen years of its existence until it has become almost an essential feature in the library of the medical man. The editor has not only continued, but has even emphasised the feature to which the "Year Book of Treatment" no doubt owes much of its success, namely the presentation of its information in the form not merely of a record, but of an appreciation of the various methods of treatment which have been introduced. To pass in review the medical literature of the year and to riddle out the chaff is a gigantic task, and the twenty-six contributors who have compressed the output of so many brains into a volume of something under 500 pages are to be congratulated on the result of their labours.

Kelly's London Medical Directory. 1897. (London: Kelly and Co. Price 6s. 6d.)

This is a very useful and complete directory, giving the names, residences, and qualifications of the medical men residing in London and the suburbs, together with their professional appointments, the medical and scientific societies of which they are members, their published works, and a selection from their contributions to periodical literature. A good deal of collateral information is also included concerning public bodies, hospitals, societies, and institutions. The only fault we have to find is the splitting up of the streets list into separate lists for each postal district, which makes the search for a street a very complex affair, especially as there is no sign at the top of the page to show which district is being dealt with. To add to the complexity certain suburbs are made to include parts of more than one postal district, and thus, to take an instance, several streets in the S.W. district are entered in the W. list merely because they are looked upon as part of West Kensington which happens to be mainly in the W. district. Multiple lists are a nuisance to all concerned, as everyone knows who, for example, uses "Churchill's Directory," and has to wade through seven separate lists before he can find out whether a man is qualified or not!

Twentieth Century Practice of Modern Medical Science. Edited by Thomas L. Stedman, M.D. Volume VII. Diseases of the Respiratory Organs and Blood, and Functional Sexual Disorders. (London: Sampson Low, Marston, and Co. 1896.)

There is no doubt that this is an interesting volume, although at the same time it must be confessed that it is somewhat of a mixture. The previous volume, after dealing with diseases of the bronchial tubes, ended with diseases of the lungs, excluding croupous pneumonia and tuberculosis. The present one begins with diseases of the pleura, then goes to asthma, hay fever, and two short chapters on diseases of the mediastinum and the diaphragm, and a long article of three hundred pages on diseases of the blood. Rachitis is then dealt with, after which are chapters on disorders of menstruation, of the male sexual organs, and finally over a hundred pages on examination of the urine. There can then be no complaint of lack of variety. The articles on "Diseases of the Pleura," by Dr. Herbert B. Whitney, and on "Asthma," by Dr. Franz Riegel, of Giessen, are very complete, and give in each case not only the opinions of their respective authors, but good *résumé*, of the views held by various authorities. The treatment of hay fever as described by Dr. Fletcher Ingals, of Chicago, illustrates the extent to which surgery intrudes on fields which used to be looked on as exclusively medical. Stress is laid upon the occurrence of hyper-sensitive spots in the nasal mucous membrane, treatment of which by means of the galvanic cautery may be required. It is probably by its effect on such sensitive patches that cocaine is so effectual in giving temporary relief. The evils of the prolonged use of this drug are, however, strongly insisted on. "It is never safe to order cocaine for a patient without writing across the face of the prescription that it must not be repeated without special order, and it is very much better for the physician to supply the remedy himself, in order that he may be sure that it is discontinued before harm results from its use." The article on diseases of the blood is full of interest to those who are prepared to take the trouble to examine it carefully according to the methods described by the author, while that by Dr. French on examination of the urine is fairly complete, but is much spoiled by the curiously imperfect woodcuts by which it is illustrated. Taken altogether, however, the volume well maintains the standard of excellence which the earlier volumes have so well established.

